# Effects of Hispidulin on the Osteo/Odontogenic and Endothelial Differentiation of Dental Pulp Stem Cells

**DOI:** 10.3390/ph17121740

**Published:** 2024-12-23

**Authors:** Yeon Kim, Hyun-Joo Park, Mi-Kyoung Kim, Hyung Joon Kim, Yong-Il Kim, Soo-Kyung Bae, Moon-Kyoung Bae

**Affiliations:** 1 Department of Oral Physiology, School of Dentistry, Pusan National University, Yangsan 50612, Republic of Koreahjoonkim@pusan.ac.kr (H.J.K.); 2 Periodontal Disease Signaling Network Research Center (MRC), School of Dentistry, Pusan National University, Yangsan 50612, Republic of Korea; skbae@pusan.ac.kr; 3 Dental and Life Science Institute, School of Dentistry, Pusan National University, Yangsan 50612, Republic of Korea; 4 Department of Orthodontics, School of Dentistry, Pusan National University, Yangsan 50612, Republic of Korea; 5 Department of Dental Pharmacology, School of Dentistry, Pusan National University, Yangsan 50612, Republic of Korea

**Keywords:** hispidulin, dental pulp stem cells, differentiation

## Abstract

**Background:** Human dental pulp stem cells (HDPSCs) with multi-lineage differentiation potential and migration ability are required for HDPSC-based bone and dental regeneration. Hispidulin is a naturally occurring flavonoid with diverse pharmacological activities, but its effects on biological properties of HDPSCs remain unknown. Therefore, we investigated the effects of hispidulin on the differentiation potential and migration ability of HDPSCs and elucidated their underlying mechanisms. **Methods:** The osteo/odontogenic capacity of HDPSCs was assessed using the alkaline phosphatase (ALP) and Alizarin Red S (ARS) staining. The migration ability of HDPSCs was evaluated using a scratch wound assay. Furthermore, the endothelial differentiation of HDPSCs was examined by using a capillary sprouting assay and by assessing CD31 expression. **Results:** Hispidulin significantly enhanced the osteo/odontogenic differentiation of HDPSCs with increased expression of osteo/odontogenic differentiation markers. Hispidulin increased the migration of HDPSCs, which was mediated by the upregulation of C-X-C chemokine receptor type 4 (CXCR4). The treatment of HDPSCs with hispidulin enhanced the differentiation of HDPSCs into endothelial cells, as evidenced by increased capillary sprouting and endothelial marker expression. In addition, we demonstrated that hispidulin activated the ERK1/2 signaling, and its inhibition by U0126 significantly suppressed the hispidulin-induced endothelial differentiation of HDPSCs. **Conclusions:** These findings demonstrate that hispidulin effectively promotes the osteo/odontogenic and endothelial differentiation, and migration of HDPSCs. These results suggest that hispidulin may have potential therapeutic applications in dental pulp regeneration and tissue engineering.

## 1. Introduction

Human dental pulp stem cells (HDPSCs) are derived from the connective soft tissue within the dental pulp chamber surrounding hard tissues [1]. HDPSCs have emerged as promising sources of mesenchymal stem cells for regenerative medicine due to their proliferative capacity, ready accessibility from extracted teeth, and remarkable multipotency [2]. Their potential for both osteogenic and odontogenic differentiation increases their value for the regeneration of mineralized tissues, such as bone and dentin [2]. Moreover, recent studies have highlighted the endothelial differentiation capacity of HDPSCs, revealing their ability to express endothelial-specific markers, such as CD31 and the von Willebrand factor, and form capillary-like sprouting structures in vitro [3]. The capacity of HDPSCs to differentiate into both osteo/odontogenic and endothelial cells is particularly significant for tissue engineering applications, enabling the simultaneous development of mineralized structures and essential vessel networks within bioengineered tissues [4,5].

Hispidulin (chemically known as 4′,5,7-trihydroxy-6-methoxyflavone) is a naturally occurring flavonoid found in various medicinal plants such as *Arrabidaea chica*, *Salvia involucrata*, and *Saussurea involucrata* [6,7]. Hispidulin has recently significant attention owing to its diverse pharmacological properties including anti-inflammatory, antioxidant, anticancer, and neuroprotective effects [8,9,10,11,12]. Some flavonoids, including isocoumarin and icariin, have been demonstrated to play crucial roles in bone metabolism by regulating osteoblast and osteoclast differentiation [13,14]. Recent studies have demonstrated that hispidulin represses bone resorption in receptor activator of the nuclear factor κB (NF-κB) ligand (RANKL)-stimulated osteoclast precursor cells and promotes osteoblastic differentiation in MC3T3E1 cells [15]. However, its effects on the biological properties of HDPSCs, including their proliferation, migration, and differentiation, remain unclear.

We investigated the effects of hispidulin on the osteo/odontogenic and endothelial differentiation of HDPSCs and elucidated the underlying mechanisms by which hispidulin has influences on the differentiation process.

## 2. Results

### 2.1. Hispidulin Accelerates the Osteo/Odontogenic Differentiation of HDPSCs

**Figure 1:**
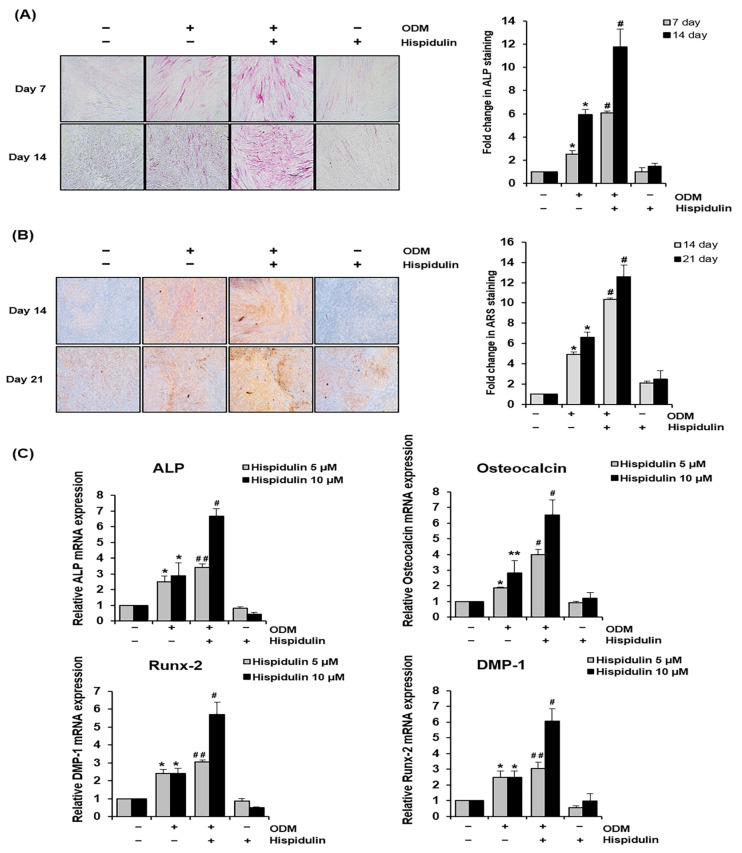
Effect of hispidulin on osteo/odontogenic differentiation and expression of osteo/odontogenic-related markers in HDPSCs. (**A**) HDPSCs were either cultured in the basic growth medium or osteogenic differentiation medium (ODM) with or without hispidulin (5 μM). ALP staining was performed on days 7 and 14. Stained cells were photographed using a phase contrast microscope at 100× magnification. ALP-positive areas were quantified by densitometry in triplicates. * *p* < 0.01 compared to control. # *p* < 0.05 compared to ODM. (**B**) The formation of mineralized nodules was evaluated by ARS staining and quantified through densitometric analysis in triplicate at days 14 and 21. The stained cells were imaged under 100× magnification. * *p* < 0.01 compared to control. # *p* < 0.01 compared to ODM. (**C**) HDPSCs were cultured with or without ODM in the presence of hispidulin for 14 days. The mRNA expression of *ALP*, *osteocalcin*, *DMP-1*, *and Runx-2* were assessed through real-time PCR analysis. All values were normalized to β-actin mRNA levels, and the expression level of the control group was designated as 1.0. * *p* < 0.01 compared to control. ** *p* < 0.05 compared to control. # *p* < 0.01 compared to ODM. ## *p* < 0.05 compared to ODM.

We first investigated the effects of hispidulin on the osteo/odontogenic differentiation of HDPSCs, for which we examined their mineralization activity using alkaline phosphatase (ALP) staining. HDPSCs cultured in the osteogenic differentiation medium (ODM) exhibited increased ALP activity compared with those cultured in the basic growth medium. This effect was significantly enhanced by hispidulin treatment, as evidenced by the increased staining intensity at both the 7- and 14-day time points (Figure 1A). Furthermore, Alizarin Red S (ARS) staining was performed to confirm the mineralization-promoting effects of hispidulin. Hispidulin treatment markedly enhanced calcium nodule formation in HDPSCs cultured in the ODM for 14 and 21 days (Figure 1B). In addition, we examined the expression of osteo/odontogenic differentiation-related markers. Real-time polymerase chain reaction (PCR) revealed that hispidulin treatment significantly upregulated the expression of ALP, osteocalcin, Runt-related transcription factor 2 (Runx-2), and dentin matrix protein-1 (DMP-1) in ODM-cultured HDPSCs (Figure 1C). Notably, treatment with 10 μM of hispidulin resulted in more pronounced effects, inducing approximately 2.3-fold, 2.3-fold, 2.4-fold, and 2.5-fold increases in the expression of *ALP*, *osteocalcin*, *Runx-2*, and *DMP-1*, respectively, compared to untreated ODM-cultured HDPSCs.

### 2.2. Hispidulin Increases the Migration Ability of HDPSCs

**Figure 2:**
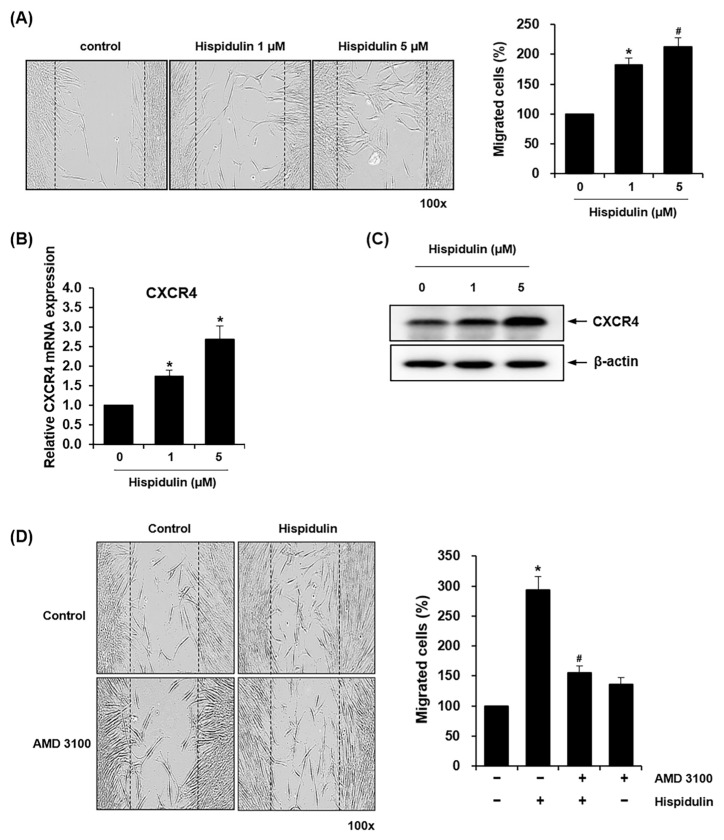
Effect of hispidulin on the migration of HDPSCs. (**A**) Scratch wound migration assays were performed on HDPSCs cultured without or with hispidulin (1 or 5 μM) for 24 h. Cell migration into the scratch wound area was photographed at 100× magnification and quantified. Results are expressed as the mean values from three independent experiments per group. * *p* < 0.05 compared with control. # *p* < 0.01 compared with control. (**B**) HDPSCs were treated with hispidulin (1 or 5 μM) for 24 h, and the expression of CXCR4 was analyzed with real-time qPCR. All values were normalized to β-actin mRNA levels, with the control group expression set as 1.0. * *p* < 0.05 compared with control. (**C**) Protein expression of CXCR4 was observed by western blotting using an anti-CXCR4 antibody (upper) and densitometric analysis (lower). β-actin was used as the loading control. (**D**) HDPSCs were incubated with 1 μM hispidulin alone or in combination with AMD3100 (50 μg/mL) for 24 h. Migrated cells beyond the reference line were photographed at 100× magnification and quantified. * *p <* 0.01 compared with control. # *p* < 0.01 compared to hispidulin.

We adopted the scratch wound migration assay to investigate the migratory capacity of HDPSCs in response to hispidulin treatment. Both 1 μM and 5 μM hispidulin treatment significantly enhanced cell migration by approximately 180% and 210%, respectively, compared to untreated controls (Figure 2A). Based on the important role of C-X-C chemokine receptor type 4 (CXCR4) in the migration and recruitment of HDPSCs [16], we examined whether hispidulin affects the expressions of CXCR4 in HDPSCs. The treatment of HDPSCs with hispidulin increased the mRNA and protein expression of CXCR4 in HDPSCs, as demonstrated by qRT-PCR and western blotting (Figure 2B,C). We treated HDPSCs with AMD3100, a specific CXCR4 antagonist, to address the involvement of CXCR4 signaling in hispidulin-induced migration [17]. As shown in Figure 2D, treatment with AMD3100 caused a 47% reduction in hispidulin-induced HDPSC migration.

### 2.3. Hispidulin Enhances the Endothelial Differentiation of HDPSCs via CD31 Expression

**Figure 3:**
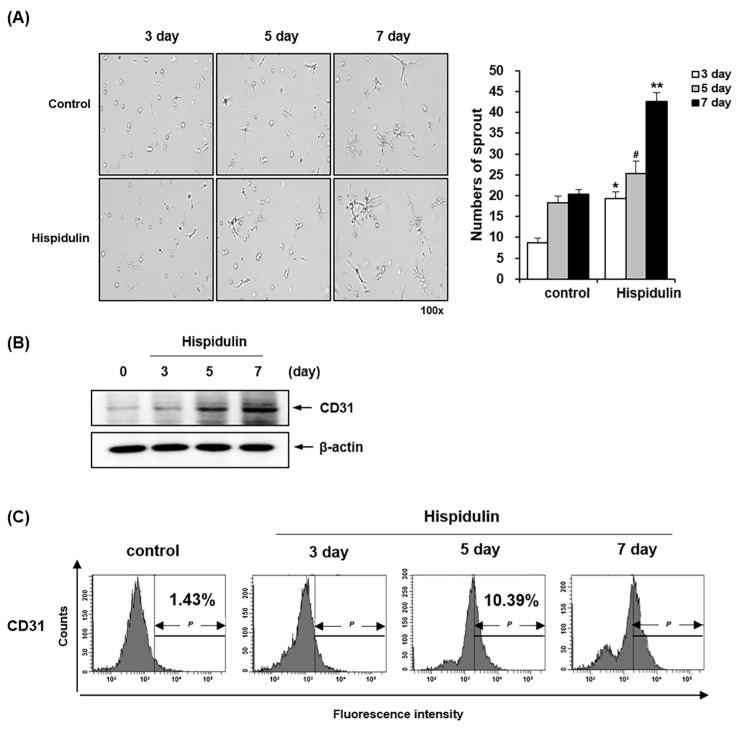
Effect of hispidulin on endothelial differentiation of HDPSCs. (**A**) HDPSCs implanted on a Matrigel-coated plate were treated with 1 μM hispidulin for 3, 5, and 7 days in the EGM-2MV. The numbers of sprouts were counted and imaged under 100× magnification. Each result represents the mean value of triplicate experiments in each group. * *p* < 0.01 compared with the 3-days control. # *p* < 0.05 compared with 5-dayscontrol. ** *p* < 0.01 compared with the 7-days control. (**B**) The protein expression of CD31 was analyzed by western blotting. β-actin was used as a loading control. (**C**) HDPSCs were treated with 1 μM hispidulin for 3, 5, and 7 days. CD31 was measured by flow cytometry.

We used a capillary sprouting assay to assess the endothelial differentiation potential of HDPSCs [18,19]. HDPSCs were cultured in an endothelial differentiation medium (EGM-2MV) supplemented with hispidulin. HDPSCs treated with hispidulin demonstrated increased capillary sprouting compared with the control group. Quantitative analysis revealed progressive capillary sprouting at days 3, 5, and 7 of hispidulin treatment, with the most pronounced effect observed on day 7 (Figure 3A). We examined the expression of the endothelial marker CD31 using western blotting and fluorescence-activated cell sorting (FACS) analysis to further elucidate the effect of hispidulin on the endothelial differentiation of HDPSCs. Western blotting demonstrated that CD31 protein expression increased in hispidulin-treated HDPSCs (Figure 3B). The cell surface expression of CD31 was further confirmed by flow cytometry to quantify the antigenic activity of proteins on the cell surface. The percentage of CD31-positive cells gradually increased from 1.43% under control conditions to 8.23%, 10.39%, and 23.17% after 3, 5, and 7 days of hispidulin treatment, respectively (Figure 3C).

### 2.4. ERK1/2 Mediates Hispidulin-Induced Endothelial Differentiation of HDPSCs

**Figure 4:**
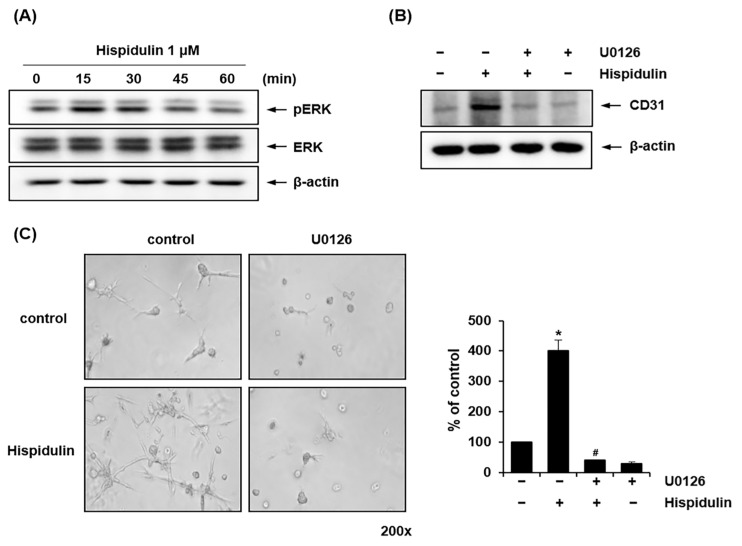
Effect of hispidulin on the ERK signaling pathway in endothelial differentiation of HDPSCs. (**A**) HDPSCs were treated with hispidulin (1 μM) for the indicated times in EGM-2MV. Cell lysates were immunoblotted with antibodies against phospho-ERK and total ERK. β-actin was used as a loading control. (**B**) HDPSCs were treated with hispidulin (1 μM) alone or in combination with U0126 (10 μM). After 7 days, CD31 protein expression was analyzed by western blotting. β-actin was used as a loading control. (**C**) HDPSCs were seeded on Matrigel-coated plates and treated with hispidulin (1 μM) alone or in combination with U0126 (10 μM) in EGM-2MV. Capillary sprouting was observed after 7 days (200× magnification). Images are representative of three independent experiments. * *p* < 0.01 compared to control. # *p* < 0.01 compared to hispidulin.

We investigated whether hispidulin treatment activated the ERK1/2 signaling pathway during the endothelial differentiation of HDPSCs. It has been previously reported that the ERK1/2 signaling plays a pivotal role in regulating the differentiation of HDPSCs into endothelial cells [20]. Western blotting revealed that the levels of phosphorylated ERK1/2 peaked 15 min after hispidulin treatment (Figure 4A). Pretreatment of HDPSCs with U0126, a mitogen-activated protein kinase (MAPK)/ERK kinase-specific inhibitor, blocked CD31 protein expression induced by hispidulin (Figure 4B). To investigate the role of ERK1/2 in hispidulin-induced endothelial differentiation of HDPSCs, HDPSCs were pretreated with U0126 before hispidulin treatment. As shown in Figure 4C, U0126 significantly suppressed hispidulin-induced sprouting formation in HDPSCs.

## 3. Discussion

Hispidulin, a naturally occurring flavone found in several medicinal plants, is a highly promising bioactive compound with a remarkable spectrum of pharmacological activities, including antioxidant, antifungal, antitumor, anti-inflammatory, and neuroprotective properties [8,9,10,11,12]. Recently, the inhibitory effects of hispidulin on RANKL-induced osteoclastogenesis and bone resorption have been demonstrated [15]. Hispidulin increased the ALP activity of MC3T3E1 cells while simultaneously inhibiting osteoclast formation in RAW 264.7 cells and bone marrow-derived macrophages (BMMs), thereby maintaining bone homeostasis [15]. We found that hispidulin promoted the osteogenic/odontogenic differentiation of HDPSCs by upregulating osteo/odontogenic-related proteins. These observations indicate that hispidulin exerts its regulatory effects on bone homeostasis by influencing both bone resorption and formation. This finding may provide a promising therapeutic potential for bone-related disorders.

Flavonoids have a remarkable ability to promote the osteogenic and endothelial differentiation of mesenchymal stem cells derived from various tissue sources, including bone marrow, adipose tissue, and dental tissues [21,22]. Naringenin, a flavanone-type flavonoid, has been reported to enhance both osteogenic and endothelial differentiation of human periodontal ligament stem cells, contributing to alveolar bone regeneration [23]. Similarly, icariin, a prenylated flavonol glycoside, has demonstrated the ability to promote bone regeneration under diabetic conditions through simultaneous activation of osteogenesis and angiogenesis in bone marrow mesenchymal stem cells [24]. Given that the coupling of osteogenesis and neovascularization is crucial for successful bone regeneration [25], our findings suggest that hispidulin may facilitate bone regeneration through its dual effects on osteogenic and endothelial differentiation.

In addition to their osteogenic/odontogenic differentiation capacity, HDPSCs possess vasculogenic differentiation potential, highlighting their promising utility in regenerative medicine and tissue engineering [26,27]. In particular, HDPSCs naturally differentiate into odontoblast-like cells at the injury site in the dental pulp to produce reparative dentin as a protective barrier [28]. In addition, HDPSCs can differentiate into vascular endothelial lineages, forming vascular networks essential for delivering oxygen and nutrients [29,30]. We observed that hispidulin promoted capillary sprouting of HDPSCs in Matrigel and increased the protein expression of endothelial cell markers, particularly CD31, in HDPSCs, suggesting a stimulatory effect of hispidulin on the endothelial differentiation of HDPSCs in vitro. Research has shown that the tooth slice/scaffold model provides a valuable in vivo approach to assess the impact of bioactive compounds, such as flavonoids and growth factors, on the differentiation potential of dental pulp stem cells [17,31,32]. Although our findings demonstrated promising effects of hispidulin on osteogenic/odontogenic and endothelial differentiation, further in vivo studies using tooth slice/scaffold models are warranted to validate these results and evaluate the functional integration of differentiated cells within the host tissue.

Our results demonstrated that hispidulin enhanced ERK1/2 phosphorylation in HDPSCs cultured in the EGM-2MVmedium, and the inhibition of MAPK/ERK kinase suppressed both, hispidulin-induced endothelial cell marker expression and hispidulin-stimulated formation of sprout-like structures in HDPSCs. The MAPK/ERK pathway plays a crucial role in mediating endothelial differentiation of different mesenchymal stem cells, including adipose tissue-derived stem cells, bone marrow mesenchymal stem cells, and stem cells from exfoliated deciduous teeth (SHEDs) [20,33,34]. Previous study has demonstrated that hispidulin treatment inhibits transforming growth factor-β1 (TGF-β1)-induced Smad2/3 signaling in cancer cell lines [35]. Furthermore, the inhibition of TGF-β signaling has been shown to enhance endothelial differentiation in SHEDs [36]. Based on these findings, we hypothesize that hispidulin may enhance endothelial differentiation of HDPSCs through the inhibition of TGF-β1-induced Smad2/3 signaling pathway. In addition, the activation of Wnt/β-catenin signaling is involved in vasculogenic differentiation of dental stem cells [37]. We are investigating whether hispidulin regulates glycogen synthase kinase-3β (GSK-3β) activity and β-catenin expression in HDPSCs during endothelial differentiation. Although our findings establish the MAPK/ERK pathway as a key mediator of hispidulin-induced endothelial differentiation, future studies are required to investigate potential interactions between hispidulin and these other critical signaling pathways, particularly the Wnt/β-catenin and TGF-β pathways, to comprehensively understand the molecular mechanisms underlying hispidulin’s effects on endothelial differentiation of HDPSCs.

**Figure 5:**
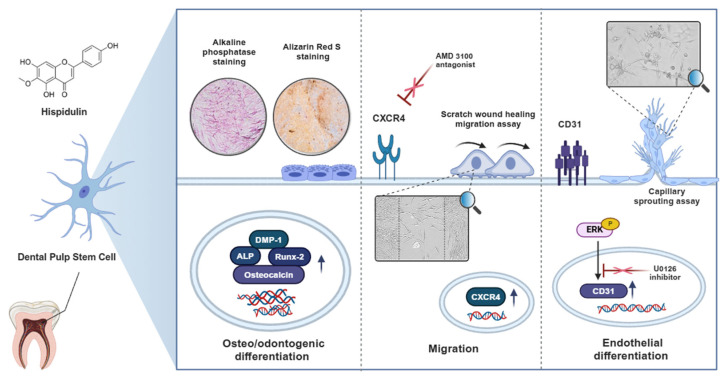
Schematic diagram illustrating the regulatory mechanisms of hispidulin in promoting osteo/odontogenic and endothelial differentiation, and migration of HDPSCs.

In conclusion, our study demonstrated the multifaceted potential of hispidulin in dental tissue engineering through its ability to enhance both the osteo/odontogenic and endothelial differentiation of HDPSCs and promote their migration. These comprehensive findings are summarized in the schematic diagram (Figure 5). While these findings suggest hispidulin’s promising therapeutic potential in for dental pulp regeneration and tissue engineering, future in vivo studies and clinical trials will be necessary to fully validate its efficacy and safety for clinical applications.

## 4. Materials and Methods

### 4.1. Reagents and Antibodies

Hispidulin, AMD3100, U0126, β-glycerophosphate disodium salt pentahydrate, ascorbic acid, dexamethasone, and methyl-thiazolyl-tetrazolium (MTT) were supplied by Sigma-Aldrich (St. Louis, MO, USA). Anti-human CD31, CXCR4, and β-actin antibodies were procured from Santa Cruz (Dallas, TX, USA), Thermo Fisher Scientific (Waltham, MA, USA), and Bioworld Technology (St. Louis Park, MN, USA), respectively. Anti-human ERK and phospho-ERK antibodies were obtained from Cell Signaling Technology (Danvers, MA, USA).

### 4.2. Cell Culture

HDPSCs were acquired from Lonza (Basel, Switzerland) and grown in α-modification of Eagle’s minimum essential medium (α-MEM, Thermo Fisher Scientific, Waltham, MA, USA), supplemented with 10% fetal bovine serum (FBS; Merck Millipore, Burlington, MA, USA), 1% penicillin-streptomycin, and 5 μg/mL plasmocin (InvivoGen, San Diego, CA, USA). HDPSCs were incubated at 37 °C in a humidified atmosphere with 5% CO_2_. For osteogenic differentiation, HDPSCs were cultured in the ODM containing 10mM β-glycerophosphate, 50 µg/mL ascorbic acid, and 0.1mM dexamethasone for 7–21days. The ODM was replaced every 2 days.

### 4.3. Cell Proliferation

The proliferation of HDPSCs was analyzed after 24, 48, and 72 h of culture growth. At the end of the culture period, cells were added in 500 μL of fresh medium containing 0.5 mg/mL MTT solution and were incubated for 4 h at 37 °C in a humidified atmosphere with 5% CO_2_. The medium was changed with 200 μL of dimethyl sulfoxide (DMSO, Sigma-Aldrich, St. Louis, MO, USA) for 3 min. Subsequently, formazan absorbance was measured at 540 nm using a microplate reader (Allsheng, Hangzhou, China).

### 4.4. ALP Staining

An ALP staining kit (Sigma-Aldrich, St. Louis, MO, USA) was used according to the manufacturer’s instructions on days 7 and 14. HDPSCs were fixed with an ALP-fixing solution, washed with distilled water, and stained with an alkaline dye mixture. Images of each sample were acquired using a Nikon ECLIPSE 55i microscope (Korea Lab Technology, Seungnam, Republic of Korea). Each experiment was performed in duplicates and three separate experiments were performed for each group.

### 4.5. ARS Staining

After osteogenic induction for 14 and 21 days, the HDPSCs were fixed with 4% paraformaldehyde for 15 min, stained with 2% ARS (Sigma-Aldrich) solution for 30 min at room temperature, and washed twice with distilled water. Images of the stained mineralized nodules were acquired in three separate wells using a Nikon ECLIPSE 55i microscope (Nikon, Minato, Tokyo, Japan) and analyzed using the ImageJ software version 1.53o to calculate the mean and standard deviation.

### 4.6. Scratch Wound Healing Migration Assay

HDPSCs were seeded in plates containing a basic growth medium until they formed an adherent monolayer. A wound line was created by scratching the plates with a 10 µL pipette tip, and the floating cells were removed twice with 10% α-MEM. After incubating the cells with 1 μM hispidulin alone or in combination with AMD3100 (50 μg/mL) for 24 h, cell migration into the scratched area was photographed using a Nikon ECLIPSE 55i microscope (Nikon, Minato, Tokyo, Japan). The average width of the gaps was calculated from images captured using a microscope at four different sites from each wound line (*n* = 4).

### 4.7. Reverse Transcription-Quantitative PCR

The total RNA was extracted with a RiboEx kit (GeneAll, Seoul, Republic of Korea) and reverse transcribed using a reverse transcription kit (Promega; Madison, WI, USA). Real-time PCR was then performed with SYBR Green premix (Enzynomics, Daejeon, Republic of Korea) using Applied Biosystems (Waltham, MA, USA). The oligonucleotide primers listed below were used: β-actin: 5′-ACTCTTCCAGCCTTCCTTCC-3′ and 5′-TGTTGGCGTACAGGTCTTTG-3′; ALP: 5′-ATTTCTCTTGGGCAGGCAGAGAGT-3′ and5′-ATCCAGAATGTTCCACGGAGGCTT-3′; osteocalcin: 5′-CAGCGAGGTAGTGAAGAGAC-3′ and 5′-TGAAAGCCGATGTGGTCAG-3′; DMP-1: 5′-AGGAAGTCTCGCATCTCAGAG-3′ and 5′-TGGAGTTGCTGTTTTCTGTAGAG-3′; Runx-2:5′-CTCTACTATGGCACTTCGTCAGG-3′ and 5′-GCTTCCATCAGCGTCAACAC-3′. The cycling conditions consisted of an initial amplification cycle at 95 °C for 10 min, followed by 40 amplification cycles at 95 °C for 15 s, 60 °C for 60 s, and 72 °C for 7 s. The expression levels of target genes were calculated using the 2^−ΔΔCt^ method, with the β-actin gene used as a housekeeping control.

### 4.8. Western Immunoblotting

Equal amounts of the samples (20 μg) were loaded on gels for sodium dodecyl sulfate-polyacrylamide gel electrophoresis (SDS/PAGE) and transferred to a nitrocellulose membrane (GE Healthcare, Chicago, IL, USA). The membrane was blocked with 5% skim milk in TBS containing 0.1% Tween-20 for 1 h at room temperature and probed with appropriate antibodies (anti-human β-actin, anti-human CXCX4, anti-human CD31, anti-human ERK, and anti-phospho-ERK) overnight at 4 °C. The signal was developed using an enhanced chemiluminescence solution (ECL; GE Healthcare, Chicago, IL, USA) and monitored using the Azure 300 Chemiluminescent Western Blot Imaging System (Azure Biosystems, Dublin, CA, USA).

### 4.9. Flow Cytometry Analysis

HDPSCs were incubated with 1 μM hispidulin for 3, 5, and 7 days. After incubation, HDPSCs were washed with PBS and treated with PBS containing PE anti-human CD31 (BD Biosciences, Bedford, MA, USA) at 4 °C. After 1 h, the cells were washed twice with PBS and analyzed by flow cytometry using a fluorescence-activated cell sorter (Becton Dickinson and Company, Franklin Lakes, NJ, USA).

### 4.10. Capillary Sprouting Assay

A sprouting assay was performed as described by Sakai et al. [18]. Before adding HDPSCs, thawed growth factor reduced Matrigel (Corning, Inc., Corning, NY, USA) was added in a 24-well plate for 60 min at 37 °C. HDPSCs were seeded on these Matrigel-coated wells and treated with 1 μM hispidulin for 3, 5, and 7 days in the EGM-2MV (Lonza, Basel, Switzerland). After the completion of sprouting, three representative images were captured from each well using a Nikon ECLIPSE 55i microscope (Nikon, Minato, Tokyo, Japan).

### 4.11. Statistical Analysis

Data are shown as the mean ± standard deviation (SD) obtained from at least three independent experiments. Statistical comparisons between groups were conducted using a one-way analysis of variance (ANOVA), followed by a Student’s *t*-test.

## Data Availability

The data presented in this study are available on request from the corresponding author.
